# Some Common SNPs of the T-Cell Homeostasis-Related Genes Are Associated with Multiple Sclerosis, but Not with the Clinical Manifestations of the Disease, in the Polish Population

**DOI:** 10.1155/2020/8838014

**Published:** 2020-11-11

**Authors:** Monika Chorąży, Natalia Wawrusiewicz-Kurylonek, Edyta Adamska-Patruno, Olga Zajkowska, Katarzyna Kapica-Topczewska, Renata Posmyk, Adam Jacek Krętowski, Jan Kochanowicz, Alina Kułakowska

**Affiliations:** ^1^Department of Neurology, Medical University of Bialystok, M. Sklodowskiej-Curie 24A, 15-276 Bialystok, Poland; ^2^Department of Endocrinology, Diabetology and Internal Medicine, Medical University of Bialystok, M. Sklodowskiej-Curie 24A, 15-276 Bialystok, Poland; ^3^Department of Clinical Genetics, Medical University of Bialystok, Waszyngtona 13 15-089 Bialystok, Poland; ^4^Clinical Research Centre, Medical University of Bialystok, M. Sklodowskiej-Curie 24A, 15-276 Bialystok, Poland; ^5^Faculty of Economic Sciences, University of Warsaw, Dluga 44/50, 00-241 Warsaw, Poland

## Abstract

**Purpose:**

Multiple sclerosis (MS) is an autoimmune disease, and genetic factors play an important role in its pathogenesis and progression. The aim of our study was to evaluate the frequencies of alleles and genetic variants of the T-cell homeostasis-related genes, in subjects with MS, as well as to investigate the association with MS clinical manifestations and disability.

**Methods:**

94 subjects with MS and 160 healthy individuals have been genotyped for seven common single-nucleotide variants in IL-2RA, CTLA4, CD40, and PADI4 genes. The ages of onset, duration of the disease, and clinical condition of the MS subjects were analysed. We used the Chi^2^ test confirmed with Fisher's exact test for statistical analysis.

**Results:**

The frequency of allele T and CT/TT genotypes (rs7093069) in the IL2RA gene, as well as the T allele and CT/TT genotypes in rs12722598, were significantly higher in the control group. The significant differences between studied groups we also found for the G allele and GG/GA genotypes of rs3087243 in CTLA4 gene, which were more common among the control group. The heterozygous genotype TC (rs1883832) of CD40 gene was more common in the control subjects, and the frequency of the alleles and genotypes in the rs1748033 of the PADI4 gene did not differ between the studied groups. Between the studied genotypes, we did not observe any significant differences in the age of onset and duration of disease, including sex stratification.

**Conclusion:**

Our results highlight the protective role of some of the T-cell homeostasis-related genetic variants in MS development, but not in its clinical manifestation.

## 1. Introduction

The immunological tolerance balance disturbances, including the presence of specific autoantibodies and the loss of function of some organs, negatively affects the health of patients with autoimmune diseases (ADs) [1]. ADs are complex disorders showing a high frequency of occurrence in the global population (~3–5%), and therefore, they are of special interest for the medical scientific community. Knowledge advancement of genetic and environmental components of the etiology of ADs may lead to establishing the basis for optimal prophylaxis, early diagnosis, and perhaps even the choice of effective treatment [2, 3]. Many GWAS studies (genome-wide association studies) provide from time to time new information about next single-nucleotide variants (SNVs), which may be the molecular risk factors or may have a protective effect for AD development and progression. These studies are based on multipopulation and multiethnic analyses of genetic variants localised in multiple loci, and they are a standard tool for molecular characterisation of the genetic background of complex diseases [3, 4]. Some of the first-described SNVs are variants within the HLA (human leukocyte antigen) region located on the short arm of chromosome 6 (6p21), which is the molecular background of many autoimmune diseases [5–7]. For example, HLA alleles of class II DRB1∗1501-DQB1∗0201 are associated with an increased risk of multiple sclerosis (MS) development, whereas HLA-DRB1∗0301-DQB1∗0201 is associated with type-1 diabetes mellitus (T1DM) [8, 9]. GWAS studies have also identified a number of genetic variants among non-HLA genes that present a significant impact on increasing AD risk, including CTLA-4 (cytotoxic T-lymphocyte-associated antigen 4), IL-2RA (interleukin 2 receptor *α*), and PTPN22 (protein tyrosine phosphatase, nonreceptor-type, 22) genes [10–13]. These candidate genes and their allelic variants are related also to T-cell activation and therefore may be involved in the pathogenesis of MS, a common demyelinating neurological disease triggered by environmental factors in genetic risk carriers [14, 15]. The CTLA-4 is a major negative regulator of T-cell responses [16], and some allelic variations in the CTLA-4 gene show the functional effects on T-cell signalling [17]. IL-2R signalling is known to be essential for regulatory T-cells, and binding IL-2 to its receptor alpha (IL-2RA) on the surface of T-lymphocytes triggers many intracellular signals, resulting in the activation and proliferation of resting T-cells, and in the generation of helper, suppressor, and cytotoxic T-cells, which are immune mediators and induce immune responses [18, 19]. Some of the single-nucleotide polymorphisms (SNPs) in or near the IL2RA gene have been already associated with the increased risk of several immune-mediated diseases, including MS [20]. CD40 is a T-cell receptor costimulator on T-cells and may make these cells effective antigen-resenting cells by upregulating cell surface molecules, which is pivotal for autoimmune disease development [21]. Further, SNPs in the CD40 gene have been linked to MS risk [22], as well as to MS progression and early disease onset in the Novosibirsk-region population [23]. Also, PADI4 was found to be extensively expressed in T-cells [24] and may affect their function [25]. Therefore, the studies investigating associations of genes that we have mentioned above with the risk of MS, considering also possible differences between populations, as well as an influence of other environmental factors that may affect and interact with a genetic background of autoimmune diseases, are of high importance. Because these genes are related to T-cell homeostasis, and MS has been described as a T-cell-mediated demyelinating inflammatory autoimmune disorder [26], we hypothesise that some of the SNVs in or near them may be related to MS development, as well as its clinical manifestations in the Polish population. Considering the above, the aim of our study was to analyse the frequencies of selected SNVs in the Polish population with MS, and their correlations with some of the clinical parameters of the disease.

## 2. Materials and Methods

### 2.1. Ethics

The study has been approved by the Bioethics Committee (Medical University of Bialystok, R-I-002/334/2018) and conducted in accordance with the Helsinki Declaration. All participants have signed informed consent forms.

### 2.2. Participants

Molecular studies were conducted in a group of 94 subjects with MS (47 males and 47 females, mean age 41.15 ± 9.74y, Table 1), which has been previously described in detail [27, 28]. The control group consisted 160 unrelated healthy adults (85 males, 75 females; mean age 37.6 ± 2.16y) with no family history of autoimmune diseases. Participants from both groups were recruited from the same geographical area and ethnic group.

### 2.3. Clinical Condition

The ages of onset and duration of the disease were analysed. The clinical condition of MS subjects was evaluated using Kurtzke's Expanded Disability Status Scale (EDSS), at the time of diagnosis, before any treatment was provided.

### 2.4. Genetic Analysis

#### 2.4.1. Genomic DNA Extraction and SNV Analysis

Genomic DNA was isolated with the Qiagen column separation method (QIAamp DNA Blood Mini Kit, Qiagen, Germany) according to the manufacturer's protocols, and then the purity and concentration of the obtained preparations were evaluated by spectrophotometric method using the NanoDrop 2000 device (Thermo Fisher Scientific, USA). All DNA samples were normalised to 50 ng/*μ*l. The SNV variants were tested in duplicates using the QuantStudio 12K Flex platform on OpenArray plates (Thermo Fisher Scientific, USA). This technology uses TaqMan molecular probes, which are applied to plates and the Genotyping Master Mix (Thermo Fisher Scientific, USA). We analysed seven SNVs in four genes: IL-2RA (rs7093069 and rs12722598), CTLA4 (rs3087243, rs231775, and rs5742906), CD40 (rs1883832), and PADI4 (rs1748033). The list of all investigated variants is presented in Table 2. We used a sample without a template as a negative control to measure any false positive signal caused by molecular contamination. The initial analysis of real-time PCR data was performed with the TaqMan Genotyping Data Analysis Software—Genotyper (Thermo Fisher Scientific).

### 2.5. Statistical Analysis

For all calculations, the Stata 15 software was used. Since our sample was small, for all the comparisons, we used the Chi^2^ test confirmed with Fisher's exact test to confirm the results. If possible, we reported the *p* value and the odds ratios (OR). *p* value below 0.05 was considered statistically significant. The Hardy-Weinberg equilibrium tests were performed for all allele frequency calculations.

## 3. Results

### 3.1. Associations between MS and Genetic Variants of the IL2RA Gene

The frequency of the polymorphic T allele of rs7093069 was significantly higher (*p* = 0.0053) in the healthy control group compared to subjects with MS (Table 2). The OR value of 0.56 indicates a strong protective effect of allele T. Similarly, the distributions of the CT and TT genotypes in the control group were significantly higher compared to participants with MS (51.88% vs. 38.8%, respectively, *p* = 0.016, OR = 0.52; and 5.0% vs. 1.06%, respectively, *p* = 0.047, OR = 0.15, Table 2).

We also observed a protective effect of allele T in rs12722598 (Table 2). The frequency of allele T was significantly higher in the control healthy group than in the group of individuals with MS (315 vs. 174 individuals, respectively, *p* = 001, OR = 0.2).

### 3.2. Associations between MS and Genetic Variants of the CTLA4 Gene

Of the three polymorphisms tested in the CTLA4 gene, our study showed significant differences in allele and genotype frequencies only for rs3087243. The frequency of allele G was higher in the group of healthy control subjects compared to the group of individuals with MS (*p* = 0.00, OR = 0.09). Similarly, a genotype distribution analysis showed significant differences in the frequency of GG and GA genotypes in the healthy control group compared to the MS group (Table 2). The GG genotype was present only in the group of healthy individuals, and OR = 0.0038 indicates an extremely strong protective effect of this genotype. The protective effect was observed for the heterozygous GA genotype, for which we have observed a significantly higher frequency in the healthy control group, compared to the group of subjects with MS (48.12% vs. 31.91%, respectively, *p* = 0.00, OR = 0.087).

### 3.3. Associations between MS and Genetic Variants of the CD40 Gene

Our analysis of the rs1883832 polymorphisms in the CD40 gene did not show any significant differences in the homozygous genotypes and allele frequencies between the studied groups. We only observed that healthy control individuals carried the heterozygous TC genotype significantly more often than the subjects with diagnosed with MS (59 vs. 23 individuals, respectively, *p* = 0.00477, OR = 0.55).

### 3.4. Associations between MS and Genetic Variants of the PADI4 Gene

We did not observe any significant differences between the studied groups in the frequencies of alleles and the genotypes of rs1748033 in the PADI4 gene (Table 2).

### 3.5. Associations between Investigated Genetic Variants and MS Clinical Manifestations

The analysis of allele and genotype frequency distribution within MS subjects did not show any significant differences between men and women (data not shown). We did not notice any associations between the studied variants, and the age of onset, the duration of the disease, or the disability status (data not shown), including sex stratification.

## 4. Discussion

The rapid technology development and novel models of collaboration between research groups have enabled to discover several genetic risk factors that have been associated with MS. However, due to the fact that the heredity of the disease has not fully been determined yet, the researchers are currently focusing on identifying alleles and genotypes, related pathways, epigenetic mechanisms, and gene-environment interactions, which may have an impact on MS development and progress [29].

In our study, we found that a T allele of the rs7093069 in the IL2RA gene was significantly more often carried by the healthy control individuals than by participants with MS, which suggests that it may have a protective effect on MS development. Also, the CT and TT genotypes distribution was significantly higher in the healthy control group subjects than in individuals diagnosed with MS, which indicates that carrying the T allele may have a strong significant protective effect against MS development. The IL2RA gene has been associated with differences in immune cell populations in subjects with MS, and Hartmann et al. found that individuals carrying the IL2RA risk alleles present a significantly increased propensity of TH (T helper) cells to develop into GM-CSF- (granulocyte-macrophage colony-stimulating factor-) producing memory TH-cells and linked this genetic risk factor with a functional feature of TH-cells in MS [30]. The immunologically relevant genes, their target proteins, and T-helper-cell differentiation have been implicated in the pathogenesis of multiple sclerosis in the European population by a collaborative GWAS of 23 research groups working in 15 different countries [31]. As investigated in our study, rs7093069 in the IL2RA gene has already been associated with some ADs, but to the best of our knowledge, our study is the first to report the association between investigated SNVs in rs7093069 and the risk of MS development.

We have also observed significant differences between healthy control subjects and individuals with MS in the allele and genotype frequencies in rs3087243 of the CTLA-4 gene. The G allele, as well as the GG and GA genotypes, was significantly more common among healthy control participants than in subjects with MS. Interestingly, the GG genotype has been identified only in the group of healthy subjects. The CTLA-4 gene has been associated with susceptibility to MS also by Kantarci et al. in the North American MS genomic screen population [32]. However, a study by Rasmussen HB et al. did not show any interaction between CTLA4 genetic variants that could be involved in the development of MS among European Caucasians [33]. A study by other authors suggests that the involvement of the CTLA4 gene in the pathogenesis of MS may be very subtle, and, rather than genetic polymorphisms that may predispose to MS development, it seems to be related to the functional changes in its pathway [34]. The discrepancies between observations may be due to the differences between studied populations and require further investigation, especially since in our studied group the GG genotype has been identified only in healthy subjects, which is intriguing. Nevertheless, when comparing results, the ethnic differences between studied populations should be always considered, as for example in our previous study, we did not observe any associations between the MTHFR mutation and the development of MS in the Polish population [35], which has been reported in the different populations [36].

Our analysis of the rs1883832 polymorphisms in the CD40 gene has shown that only the heterozygous TC variant is more common among healthy individuals compared to subjects with MS, without any differences between homozygous variant frequencies. Although the T allele of rs1883832 has been previously associated with increased susceptibility to MS [22], MS development, age of onset, and progression, including in the Polish population [37], we did not observe this association in our studied group. This could be due to the small study sample in our analysis, but also the regional differences cannot be excluded. Moreover, the importance and role of the CD40 gene variants in MS development is not clear and still under investigation. A lower expression of CD40, being a costimulator of T-cell activation, has been associated with increased risk of MS development, despite that the same CD40 variant may be associated with the reduced risk of other inflammatory ADs, and could be consistent with other, for example immunoregulatory, functions of CD40, playing an important role in protection from MS development [38].

In our study population, we did not notice any significant differences between cases and control group in the frequencies of alleles and genotypes of rs1748033 in the PADI4 gene. Our results are in line with the study by Tommasi et al. [39]. The role of PADI4 isoforms in some ADs has been studied extensively, and even if in MS the extensive deamination of brain proteins is observed in active lesions, the role of the PADI4 gene in susceptibility to MS development has not been described so far [40].

Also, we did not notice any crucial differences between studied genotypes in the clinical manifestations of MS that could be explained by the limited samples described herein. Moreover, the cerebral activity and connectivity of neural circuits responsible for locomotor functions, that are included in the EDSS scoring, depend also on the other factors that we have not investigated in our study, such as serum concentrations of vitamin D [41], which is involved in the immune responses and brain functions [42] and is considered a serum biomarker in neurological diseases as well [43]. The risk, progression, and clinical manifestation of MS has been shown to be associated also with the other SNVs [44] that we have not analysed in our study and might have an impact on our results. Nevertheless, the small study sample is a major limitation of our study; therefore, our results should be interpreted with caution, and further investigations and observations in the larger population are required.

## 5. Conclusions

In summary, despite the relatively small study group, our results show that some of the investigated SNVs within the IL2RA, CTAL4, and CD40 genes are more common in MS individuals, and others may have a protective role, indicating the need and importance of further investigation.

## Figures and Tables

**Table 1:** The clinical characteristic of MS subject group.

Characteristics	MS subjects, *n* = 94	Women, *n* = 47	Men, *n* = 47
Age at onset (years)	41.14 ± 0.79	42.78 ± 0.98	37.14 ± 1.11
Disease duration (years)	8.12 ± 0.42	8.18 ± 0.51	7.96 ± 0.75
EDSS	1.85 ± 0.10	1.84 ± 0.12	1.87 ± 0.19

Data is presented as mean ± SD. MS, multiple sclerosis; EDSS, Expanded Disability Status Scale.

**Table 2:** The genotypes and allele frequencies in the studied groups.

Gene	SNV	Genotypes/allele	MS (*n* = 94)		HC (*n* = 160)		*p*∗	OR (95% CI)
			*n*	Frequency	*n*	Frequency		
*IL2RA*	rs7093069	CC	57	(60.64%)	69	(43.12%)	NS	
		CT	36	(38.8%)	83	(51.88%)	0.016	0.52
		TT	1	(1.06%)	8	(5.0%)	0.047	0.15
		C	150	(0.80)	221	(0.69)	0.0053	0.56
		T	38	(0.20)	99	(0.31)		
	rs12722598	TT	80	(85.11%)	156	(97.5%)	NS	
		TC	14	(14.89%)	3	(1.88%)	0.06	4.2
		CC	—		1	(0.62%)	NS	
		T	174	(0.93)	315	(0.99)	0.001	0.2
		C	14	(0.07)	4	(0.01)		

*CTLA4*	rs3087243	GG	0	(0.00%)	69	(43.12%)	0.00	0.0038
		GA	30	(31.91%)	77	(48.12%)	0.00	0.087
		AA	64	(68.09%)	14	(8.75%)	NS	
		G	30	(0.16)	215	(0.67)	0.00	0.09
		A	158	(0.84)	105	(0.33)		
	rs231775	AA	36	(38.30%)	51	(31.88%)	NS	
		AG	45	(47.87%)	82	(51.25%)	NS	0.78
		GG	13	(13.83%)	27	(16.88%)	NS	0.68
		A	117	(0.62)	184	(0.58)	NS	
		G	71	(0.38)	136	(0.43)		
	rs5742906	CC	71	(75.53%)	109	(68.13%)	NS	
		CT	20	(21.28%)	40	(25.00%)	NS	0.77
		TT	3	(3.19%)	11	(6.88%)	NS	0.41
		C	162	(0.86)	258	(0.81)	0.069	
		T	26	(0.14)	62	(0.19)		

*CD40*	rs1883832	TT	7	(7.45%)	10	(6.25%)	NS	
		TC	23	(24.47%)	59	(36.88%)	0.0477	0.55
		CC	64	(68.09%)	91	(56.88%)	NS	0.99
		T	37	(0.20)	79	(0.25)	NS	
		C	151	(0.80)	241	(0.75)		

*PADI4*	rs1748033	TT	7	(7.45%)	13	(8.13%)	NS	
		TC	45	(47.87%)	58	(36.25%)	0.068	1.64
		CC	42	(44.68%)	89	(55.63%)	NS	1.14
		T	59	(0.31)	84	(0.26)	NS	
		C	129	(0.69)	236	(0.74)		

∗For genotypes, we report *p* value for Chi^2^ test, and for alleles, we report Fisher's exact *p* value. SNV, single-nucleotide variant; MS, multiple sclerosis; HC, healthy control; OR, odds ratio.

## Data Availability

The data used to support the findings of this study are available from the corresponding author upon request.
